# Analysis of Physical and Mechanical Properties of the Mortar in the Historic Retaining Wall of the Gediminas Castle Hill (Vilnius, Lithuania)

**DOI:** 10.3390/ma12010008

**Published:** 2018-12-20

**Authors:** Bronius Jonaitis, Valentin Antonovič, Arnoldas Šneideris, Renata Boris, Robertas Zavalis

**Affiliations:** 1 Department of Reinforced Concrete Structures and Geotechnics, Faculty of Civil Engineering, Vilnius Gediminas Technical University, Saulėtekio al. 11, LT-10223 Vilnius, Lithuania; bronius.jonaitis@vgtu.lt (B.J.); robertas.zavalis@vgtu.lt (R.Z.); 2 Institute of Building Materials, Faculty of Civil Engineering, Vilnius Gediminas Technical University, Linkmenų g. 28, LT-08217 Vilnius, Lithuania; valentin.antonovic@vgtu.lt (V.A.); renata.boris@vgtu.lt (R.B.)

**Keywords:** historic retaining wall, historical lime mortar, microstructure, X-ray diffraction, calcium-silicate-hydrate (C-S-H), mechanical properties

## Abstract

This study analysed the results of the joint research into the building materials of the historic masonry of the retaining wall of the Gediminas Castle Hill dating back to the end of the 13th and beginning of the 14th centuries. Core samples for material testing were collected along the entire height of the retaining wall to assess its load bearing capacity. Boreholes were drilled 600–1000 mm from the exterior of the wall to determine the properties of the ceramic bricks and lime mortar that were not affected by external factors. The analysis of the microstructure and chemical composition, mechanical and physical properties of the ceramic bricks and lime mortar is presented. The high compressive strength (8.5 MPa) of the lime mortar was the result of using hydraulic lime and crumbs of ceramic bricks as mortar aggregate.

## 1. Introduction

Historic masonry structures degrade over time due to humidity and temperature fluctuations. Cyclic exposures to humidity and frost as well as environmental chemical pollutants destroy the brickwork and mortar joints. Depletion of mortar in the horizontal joints deteriorates the bond between the mortar and bricks. Thus, the cross-sections of masonry constructions, as well as their load bearing capacities, diminish. As a rule, the aim of the investigation of historic buildings and structures is not only to identify the construction materials used and to evaluate their current conditions but also to propose measures for the preservation and structural strengthening of the buildings.

Restoring damaged historic brickwork involves repairing the depleted joints and replacing the damaged units with new ones. Damaged bricks are replaced by new bricks of identical size and colour while the joints are repaired using mortar of the same colour. It is very important to use materials with similar physical and mechanical properties to those of the materials originally used in the historic buildings. The similar properties of the materials used for restoration allow to avoid the damage caused by the different deformation characteristics of the new materials. The strength and deformation characteristics of the mortar used to repair brickwork joints must be the same as those originally used in the building under renovation [1,2,3,4,5]. The mechanical properties of the mortar depend on the type of binding material, its amount, chemical composition, aggregate particle size distribution and particle surface, ratio of the binding material and aggregates, and admixtures. Lime mortar was widely used for historic masonry structures in the past as it was considerably easy to manufacture and inexpensive. Lime had been known in Lithuania since the 13th century [6] when brick and lime mortar manufacturing began. Lime mortars were used for binding bricks and stones. Today, such mortars are found in the remains of historical masonry, i.e., castles, fortresses, and retaining walls.

In the past, lime mortar was made of lime, various aggregates (most often sand), and water. The mechanical properties of the mortar depended on the type of lime. Both hydraulic and non-hydraulic lime was used for mortar manufacturing. Non-hydraulic lime resulted in ancient mortars with low compressive strength. The strength of such mortars was only 0.5–3.0 MPa [7]. The mechanical properties of historic mortars obtained from hydraulic lime were superior to those of mortars obtained from non-hydraulic lime. Investigations into the properties of the mortar used in the Alhambra Palace (Spain, 13th century) showed that its compressive strength was 11–15 MPa [8].

Other sources stated that the compressive strength of mortars obtained from hydraulic lime can be quite low depending on the materials used and other factors. A study investigating the ancient brickwork in Becin (Turkey, 14th century) reported the compressive strength of hydraulic lime mortar to be 1.00–3.86 MPa [9].

Pozzolanic admixtures (tuff, volcanic rock, ash, etc.) improve the hydraulic and mechanical properties of lime mortars. The reactions of lime and pozzolanic admixtures produce calcium silicate hydrate (C-S-H) in the mortar, densify the structure of the material, increase the strength of the mortar, and reduce its solubility. Crushed or ground fired clay added to the materials causes the additional formation of C-S-H and calcium aluminium hydrosilicates (C-A-S-H). Černý et al. [10] analysed the use of lime mortars for the restoration of historic structures and found that the compressive strength of the mortar containing pozzolanic admixtures was 3–4 times higher than the compressive strength of lime mortar without any admixtures, which was 1.11 MPa after 28 days of curing. 

Investigating the mortars of historic buildings revealed that aggregates of crushed ceramic bricks fired at low temperature were used to prepare mortars. Testing such mortars containing crushed brick aggregates showed that additional aluminosilicate products were generated in the reaction between lime and aggregates [11].

The particle size distribution of the aggregate also has an effect on the mechanical properties of lime mortar. Stefanidou and Papayianni [1] stated that lime mortars containing fraction aggregates 0–4 mm in size had the highest strength compared to mortars containing fraction aggregates 0–2, 0–8, or 0–16 mm in size.

It should be noted that the analysis of lime used in historic mortars is more complicated due to the variety of their physical and chemical characteristics [12,13]. The following test methods are typically used for chemical and mineralogical characterisation of historic mortars: X-ray diffraction (XRD), scanning electron microscopy (SEM), and thermal analyses (TA) [14,15].

Two processes occur during the hardening of hydrated lime: the crystallisation of portlandite (Ca(OH)_2_) and the binding of atmospheric CO_2_ and carbonation of Ca(OH)_2_ [15,16]. Calcium carbonate (calcite, vaterite, or aragonite) is the final product in mortar hardening. Using the test methods mentioned above, calcite was found to be the major mineral, which is easily identified, in historic mortars. The C-S-H formed when using pozzolanic admixtures is an entirely amorphous phase, which is difficult to identify using XRD testing. Other minerals, such as quartz, gypsum, dolomite, muscovite, feldspar, etc., are often identified in tested historic mortars [8,17,18].

Bricks and binding materials used in historic masonry structures were produced by craftsmen. Bricks and binding materials differed and were specific for their respective historic periods. Thus, it is important to accumulate the largest possible amount of information on the composition and mechanical properties of building materials used in masonry structures during different historic periods. Investigating historic brickwork must provide information about the composition of the mortar used so that alternative materials can be found or the ingredients of the mortar can be reconstructed [5,19].

The oldest masonry structures in Vilnius date back to the 13th–14th centuries. The retaining wall of the Gediminas Castle Hill (Vilnius, Lithuania) is one of the oldest masonry structures dating back to the end of the 14th–beginning of the 15th centuries [6]. The aim of our study was to conduct an integrated investigation into the brickwork of the remains of the retaining wall of the Gediminas Castle Hill: masonry units (bricks), mortar, microstructure of the mortar, its chemical composition, and its physical and mechanical properties.

## 2. Retaining Wall of the Gediminas Castle Hill

**Figure 1:**
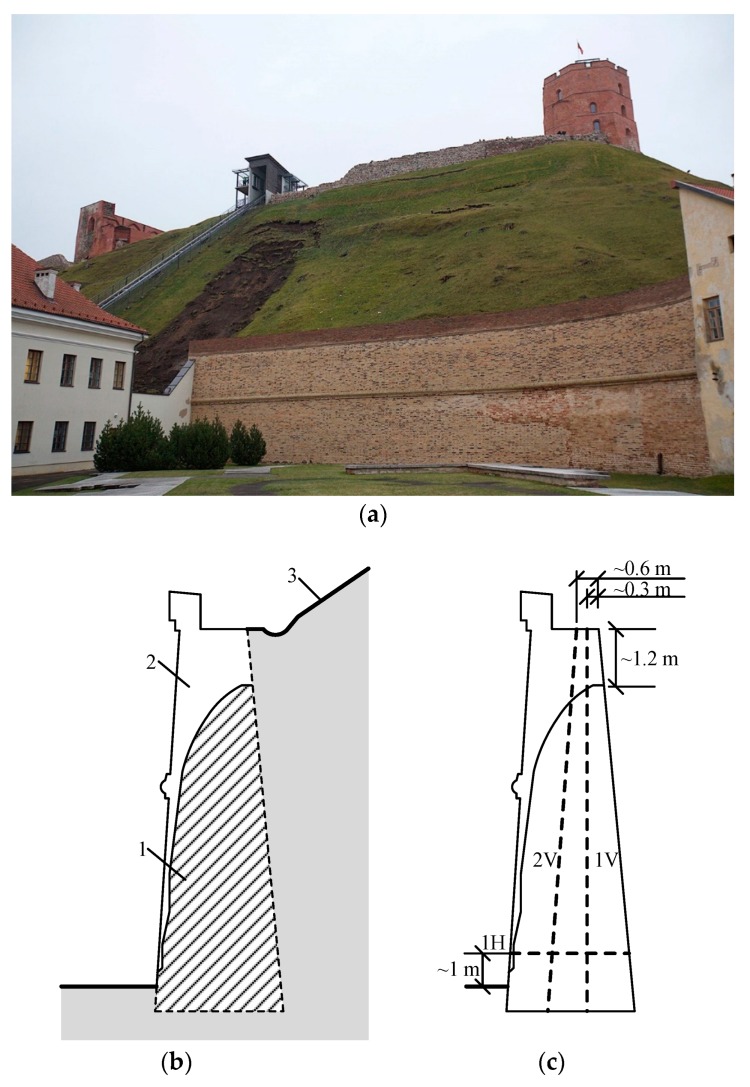
Restored Gediminas Castle Hill retaining wall and its cross section [20]: (**a**) restored Gediminas Castle Hill retaining wall; (**b**) wall section: 1 is the remaining part of the wall, 2 is the restored part of the wall, and 3 is the surface of the hill; and (**c**) locations of boreholes (1V, 2V, and 1H).

The retaining wall of the Gediminas Castle Hill is a gravity wall of variable thickness. The length of the wall leaning into the hill is approximately 42 m. The thickness of the wall is 3.2 m at the bottom and 2.8 m at the top. The height of the wall including the parapet is approximately 9 m [20]. The section drawing of the retaining wall is presented in Figure 1 [6]. The retaining wall was restored in 1962 according to the design of architect Sigitas Lasavickas [20].

**Figure 2:**
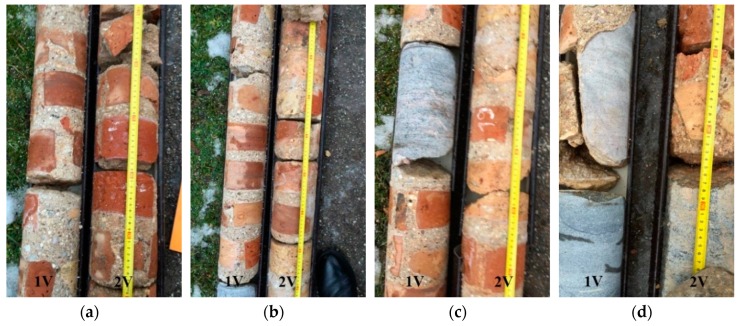
V1 and V2 bore samples. Depth of the borehole for the samples: (**a**) 2.6–3.0 m, (**b**) 3.0–3.7 m, (**c**) 3.7–4.2 m, and (**d**) 5.7–6.0 m.

**Figure 3:**
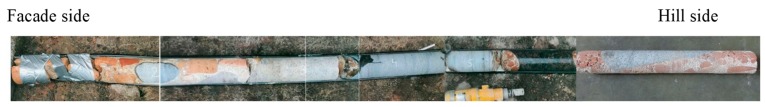
Structure of the horizontal section of the wall (borehole 1H).

Two vertical boreholes (1V and 2V) were drilled approximately 9 m deep along the entire height of the wall (pictures of the core samples are illustrated in Figure 2) and one horizontal borehole (1H) was drilled along the entire width of the wall (Figure 3).

Borehole 1H was drilled 1 m above the ground surface (Figure 1c).

Borehole 1H revealed that the retaining wall consists of layers; the external layers (500 mm wide) consists of ceramic bricks of different colours and the inner layer consists of field stones. The 1V and 2V boreholes were drilled 300 and 600 mm, respectively, from the side of the wall that is in contact with the ground. The analysis of the core samples revealed that the inner layer of the wall consisted of boulders, mortar, and ceramic bricks of different colours (Figure 2 and Figure 3). The gaps between the bricks and boulders were filled with lime mortar. The thickness of the mortar between the joints reached up to 20 mm. The lower part of the wall (~5 m deep) contains larger size boulders. In some places, the gaps between boulders were up to 100–120 mm. These gaps were filled with mortar and different size wrecked bricks. That type of mortar could be called coarse aggregate concrete.

The mortar consisted of lime and coarse sharp sand. The sand contained coarse grains 3–10 mm in size. In some places the mortar had a brownish colour, apparently resulting from the presence of clay particles. Crumbles of bricks and inclusions of unmixed lime were also found in the mortar. A similar mortar structure was also found in other brick buildings in Vilnius dating from the same period [6]. According to Böke, et al. [11], who researched historic mortars, crushed bricks were added to hydraulic mortars to increase their pozzolanicity. Levandauskas identified the white stripes seen in historic mortars as lime inserts, and presumed that this white colour was caused by pieces of non-hydrated lime [6]. The non-hydrated lime pieces might have been used to reduce the water demand in the mortar mix. The lime pieces could not only dehumidify the mortar, but also generate heat, which, in turn, could accelerate the hardening of the lime mortar.

**Figure 4:**
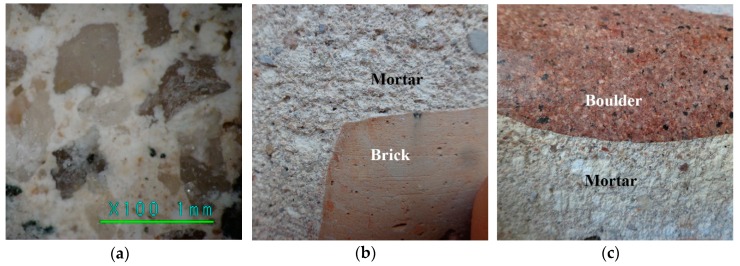
Contact zone between bricks, boulders, and mortar: (**a**) aggregates and lime mortar, (**b**) lime mortar and bricks; and (**c**) lime mortar and boulders.

The analysis of the core samples revealed good contact between the binding material and aggregates and very good coverage of the aggregate particles within the binding material layer. This can be seen from picture done with portable digital microscope DG-3x (Scalar Corp., Tokyo, Japan). in Figure 4a. The same can be said about the quality of the contact between mortar and bricks (Figure 4b), and mortar and boulders (Figure 4c) from pictures taken by a Sony digital camera (Sony Corp. DSC-W730, Shenzhen, China).

## 3. Specimens and Test Methods

**Figure 5:**
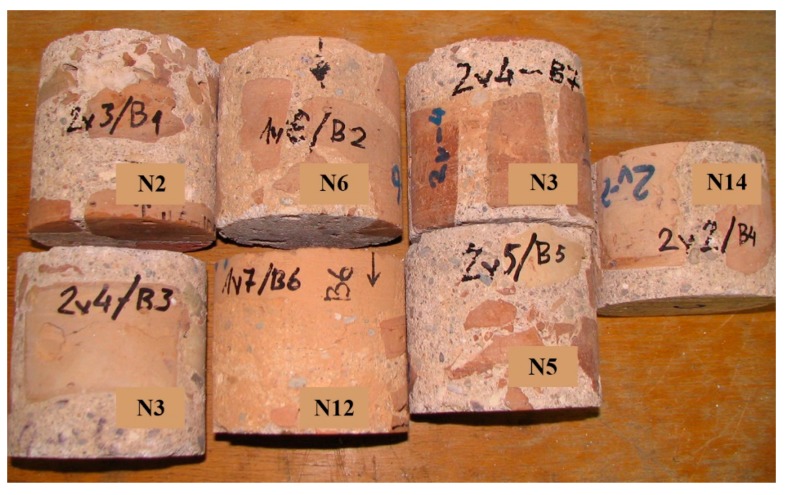
Specimens for compressive strength testing.

Test specimens of mortar containing brick crumble aggregates, bricks and mortar (Figure 5) were cut from the core samples (Figure 3). The compressive strength of the mortar containing brick crumble aggregates was measured by testing specimens 115 mm in diameter and 115 mm in height (Figure 5) according to LST EN 12504-1 standard [21], while the density was measured according to LST EN 12390-7 standard [22].

The average compressive strength of the ceramic bricks was measured by testing 50 × 50 × 50 mm size specimens according to LST EN 772-1 standard [23]. The normalised average compressive strength of the ceramic bricks was calculated from the average compressive strength by applying the shape factor *d*. The water absorption of the bricks was measured according to LST EN 772-21 standards [24].

The physical and mechanical properties of the mortar were measured by testing 40 × 40 × 40 mm cubes cut at different depths of the core samples. The compressive strength of the mortar was measured through tests according to LST EN 1015-11 standard [25] and the density was measured according to LST EN 1015-10 standard [26].

The particle size distribution of quartz sand and the amount of sand and lime in the mortar were measured during the tests. Sand was segregated from the mortar according to the methodology described by Levandauskas [6]. For the particle size distribution test, the mortar was crushed and the binding material (lime) was dissolved in 2N HCl. The solution was sieved through a 0.005 mm sieve and filtered. The precipitate on the filter and the residue on the sieve were dried to constant mass. The sizes of the sand fractions and the fineness modulus of the sand were measured by sieving the sand through a standard set of sieves (2.5–0.14 mm).

The qualitative analysis of the phase composition was performed using a DRON-7 X-ray diffractometer (Bourevestnik, St. Petersburg, Russia). A graphite monochromator (Bourevestnik, St. Petersburg, Russia) was used to obtain the X-ray emission lines of the Cu Kα complex (λ = 0.1541837 nm). The test parameters were as follows: anode voltage 30 kV, anode current 12 mA, diffraction angle 2θ range 5–60°, detector step 0.02°, and intensity measuring span 0.5 s. The phases were decoded from the XRD patterns using the International Centre for Diffraction Data (ICDD) diffraction database.

Microanalysis of the materials was conducted using a JOEL JSM-7600F SEM instrument equipped with an Inca Energy 350 (Oxford Instruments, High Wycombe, UK) energy dispersive X-ray spectrometer (EDS). The parameters of the SEM and EDS tests were as follows: voltage 10 kV; distance to specimen surface 10 mm; and magnification 1000×, 5000×, and 10,000×. Before testing, the specimens were covered with an electrically conductive thin Au layer by evaporating an Au electrode in vacuum using a Quorum Q150R ES instrument (Quorum, Laughton, UK).

Analysis of the chemical composition of the specimen was performed using wavelength dispersive X-ray fluorescence spectroscopy (WDXRF). An AxiosmAX (PANalytical, EA Almelo, the Netherlands) spectrometer with a 4 kW X-ray tube and a Rh anode were used for the test. Test results were processed using the Omnian (PANalytical) trendsetting standardless analysis package. Specimens for the tests were prepared by grinding the sample to micron-size particles (for 5 min in a rotation mill at 550 rpm) and pressing the powder into 37 mm diameter tablets. Chemical analysis was performed using dry matter, and the carbon content was not tested.

## 4. Analysis of the Test Results

### 4.1. Physical Mechanical Properties of Brickwork Components

The density and compressive strength of the mortar containing coarse brick chip aggregates was measured by testing 115 mm diameter cylinders cut from the core samples obtained from different depths. The average density of the tested specimens was 1720 kg/m^3^ (min. value 1650 and max. value 1780 kg/m^3^, Table 1); the average compressive strength was 10.2 MPa (min. value 7.4 and max. value 12.7 MPa). The minimum and maximum values for the compressive strength were recorded in specimens obtained from core samples N2 (borehole 1V) and N12 (borehole 2V), respectively.

Visual examination revealed that solid moulded bricks were used for the brickwork. The average normalised compressive strength (Table 2) of the brick specimens collected at different depths of the masonry structure was *f_b_* = 20.5 MPa (variation 33.5%). The water absorption and volumetric density of the bricks were 16% and 1835 kg/m^3^, respectively (Table 2). The density and water absorption values were similar to the corresponding values for solid ceramic bricks from the same strength class (average normalised compressive strength 20 MPa) used in constructions at the present time. Water absorption of modern bricks is 6–21%, while the volumetric density is 1500–2400 kg/m^3^.

Data on the particle size distribution, grain size, and type of grain surface of quartz sand, and the possible sand to lime ratio in the mortar were determined through mortar tests. 

The particle size distribution and fineness modulus (*FM* = 2.75) of sand were determined by sieving the sand through a standard set of sieves. The largest sand grains had a diameter of approximately 12–13 mm. The surfaces of the grains were rough and their edges were sharp. The particle size distribution of sand in historical mortar meets the requirements for granulometric composition of sand used in mortars nowadays. The lime and sand mass ratio in the mortar is 1:2.3.

The water absorption, density, and compressive strength of the mortar were determined by testing specimens cut from core samples collected from different depths. The obtained results are presented in Table 3. The average measured water absorption was 15.7% (variation 8.1%), which indicated that the mortar had a rather uniform structure. This was confirmed by the bulk density of the mortar. The average bulk density of the mortar was 1560 kg/m^3^ (variation 6.8).

**Figure 6:**
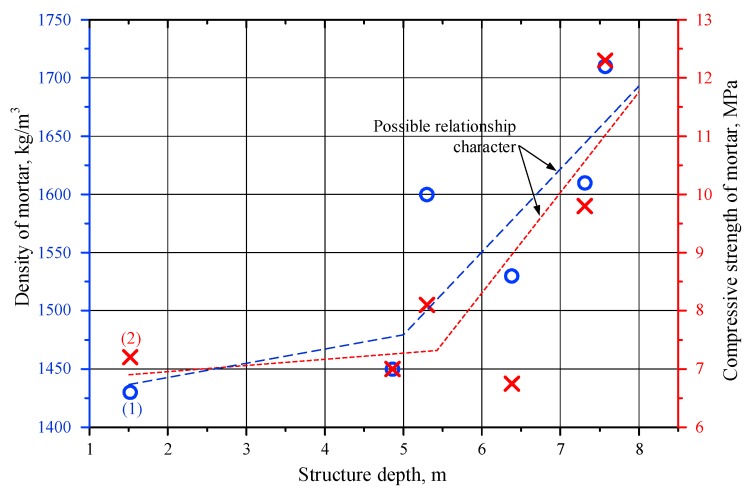
Relationship between mortar density and structure depth (1), and compressive strength and structure depth (2).

The average compressive strength of the mortar was 8.5 MPa (variation 25%). Mortar specimens cut from core sample N11 (borehole 2V) obtained from a 7.31–7.57 m depth had the highest compressive strength of 12.3 MPa. Tests of mortar specimens cut from core samples obtained from different depths revealed that the density and compressive strength of the mortar from the deeper layers were higher (Figure 6). We observed an increasing trend for the density and compressive strength of the mortar as the collection depth of the sample increased.

The compressive strength of the lime mortar in the investigated retaining wall of the castle hill was adequately high and reached values of up to 8.5 MPa. The average compressive strength of the mortar containing brick crumbles was 10.2 MPa. A low variation was observed for the strength and density of lime mortar and lime mortar containing wrecked bricks (Table 1, Table 2 and Table 3). These findings proved that high quality materials were used in the historic masonry structure.

Mechanical properties of mortars are known to be influenced by the structure of the mortar, chemical composition and degree of lime hydration, composition of aggregates, and the contact between the binding material and aggregate grains. Microanalysis of mortar components and structure was performed to determine the above-mentioned factors. The obtained results are discussed in Section 4.2.

### 4.2. Mortar Component Tests

The chemical composition of the fine fraction of the mortar was tested using X-ray fluorescence spectroscopy (XRF) analysis, and the results are presented in Table 4. The results revealed that the oxide composition of the mortar was similar along the entire height of the historical brickwork. The content of the major oxides was determined as follows: CaO: 21.6–30.7, SiO_2_: 41.6–53.2, Al_2_O_3_: 4.0–5.2, MgO: 6.0–10.8 and Fe_2_O_3_: 1.0–1.7 wt.%.

**Figure 7:**
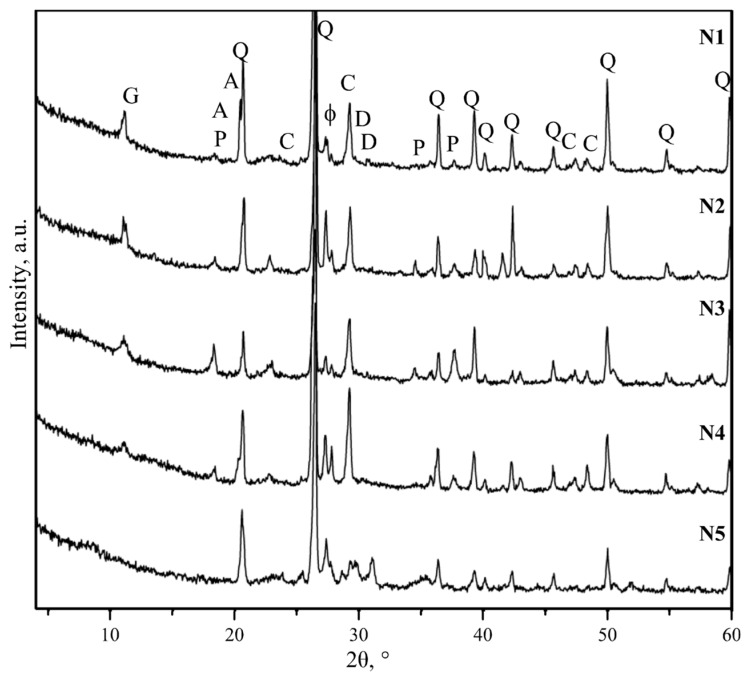
Diffraction curves of tested specimens (N1–N5): C—calcite, Q—quartz, ɸ—feldspar, G—gypsum, P—portlandite, A—gibbsite, and D—dolomite.

The qualitative phase analysis of the mortar (Figure 7) showed that the same minerals were clearly identified in all specimens: quartz (SiO_2_), calcite (CaCO_3_), portlandite (Ca(OH)_2_), gypsum (CaSO_4_∙2H_2_O), feldspars (KAlSi_3_O_8_–NaAlSi_3_O_8_–CaAl_2_Si_2_O_8_), gibbsite (Al(OH_3_)), and dolomite (CaMg(CO_3_)_2_). Weakly expressed amorphous hump between about 20° and 30° 2θ also observed in all samples. This hump apparently indicates the presence of poorly crystalline calcium silicate hydrate phase (C-S-H) [27].

**Figure 8:**
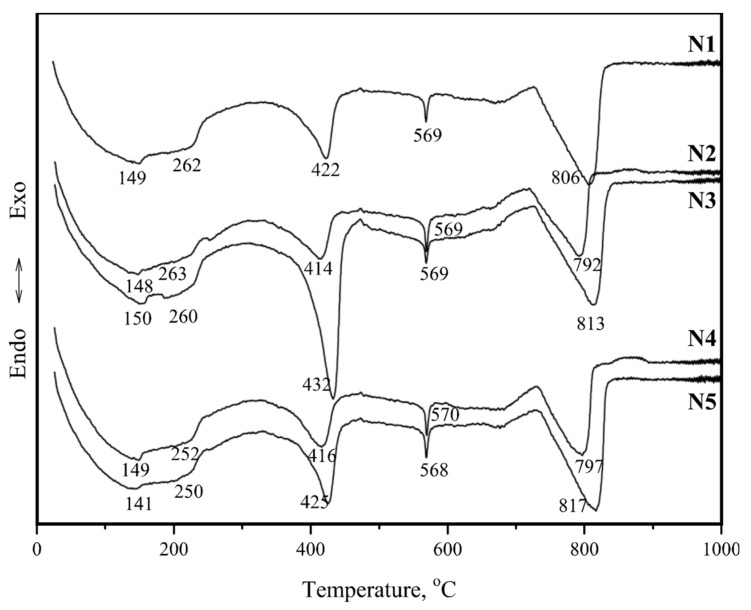
DTA curves of tested specimens (N1–N5).

Figure 8 illustrates the differential thermal analysis (DTA) curves of the mortar specimens. The weight loss values in different temperature ranges obtained from the thermogravimetric (TG) curves are presented in Table 5. All tested specimens produced five endothermic peaks in the 140–150, 250–263, 410–430, 568–570, and 790–820 °C temperature ranges (Figure 8). 

According to Moropoulou, et al. [28], the endothermic peak at approximately 100 °C was related to the removal of the physically absorbed water. The endothermic peak at 120–200 °C was characteristic for gypsum dehydration. The first endothermic peak at 140–150 °C might be seen as the total (cumulative) effect corresponding to the removal of hygroscopic water and gypsum dehydration. The weight losses of the specimens (Table 5) indicated that approximately 1% of the hygroscopic water was removed from the specimens in the 50–120 °C temperature range and approximately 1% was removed in the 120–200 °C range, apparently due to gypsum dehydration.

Chemically-bound water was removed (4.6–7.6%) in the 200–600 °C temperature range (Table 5). According to Ukrainczyk, et al. [29] and Cardoso, et al. [30], the second endothermic peak at 250–263 °C could be attributed to the dehydration of gibbsite. The third endothermic peak at 410–430 °C occurred due to the dehydration of portlandite, which usually takes place at 400–520 °C [31]. Lastly, the fourth endothermic peak at 570 °C could be attributed to the α → β phase transition of quartz [32,33].

At temperatures below 600 °C, a weight loss up to 5.6–8.9% was observed in the specimens (Table 5). This weight loss was partially attributed to the disintegration of carbonate compounds (calcite and dolomite) and the release of CO_2_ (the fifth endothermic peak at approximately 790–820 °C) [34].

It is known that carbonation in lime mortar is only possible when CO_2_ can easily penetrate the mortar and there is enough water in the material for the dissolution of CO_2_ and Ca(OH)_2_ [35]. The results presented in Table 5 indicates that the wall was affected by different environmental conditions regarding the wall height. Dehydration of low crystallisation phases, such as C-S-H and C-A-S-H, occurred at 200–600 °C. 

**Figure 9:**
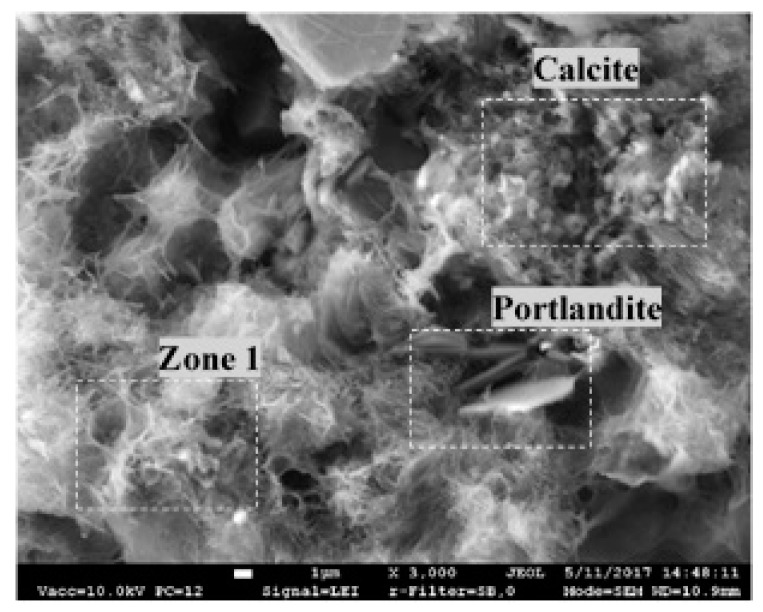
Representative image of the binding material in the tested mortar specimens.

A representative image of the binding material in all specimens obtained by SEM analysis is presented in Figure 9. The image reveals the prevalence of rhomboid-shaped calcite minerals, and fibre-shaped low crystalline C-S-H or C-A-S-H (Zone 1). Plate-shaped formations typical for portlandite are also observed.

Different aggregates were visually identified in the core samples: sand grains, brick crumbles 5–30 mm in size, and occasional white inclusions 5–10 mm in size. X-ray microanalysis revealed that the white inclusions (in specimens N1 and N2) were composed of O, Si, Al, Ca, Mg, and C (Table 6). 

**Figure 10:**
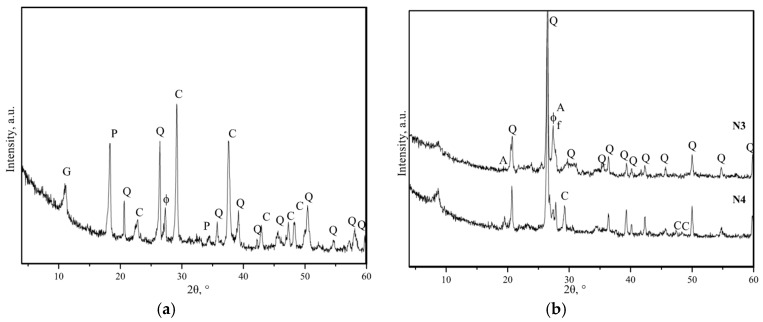
Diffraction curves of aggregates in the mortar: (**a**) white inclusions and (**b**) ceramic brick crumbles: C—calcite, Q—quartz, ɸ—feldspar, G—gypsum, P—portlandite, A—gibbsite, and D—dolomite.

Calcite, quartz, feldspars, gypsum, and portlandite were identified by subjecting the specimens to diffraction analysis (Figure 10a). The results suggested that the white inclusions in the mortar could have been caused by lime lumps (as well as previously defined in historic buildings [6]).

The ceramic crumbles in the mortar had different colors: some pieces of ceramic were light while others were dark. Comparing the test results (Table 6) with the chemical composition of clays extracted from various Lithuanian quarries described by Kubiliūtė and Kaminskas [36], it can be considered that brick crumbles in the tested mortar originated from local clays. According to studies on Lithuanian clay [37], the colour of the final ceramic products depends on the firing temperature. 

**Figure 11:**
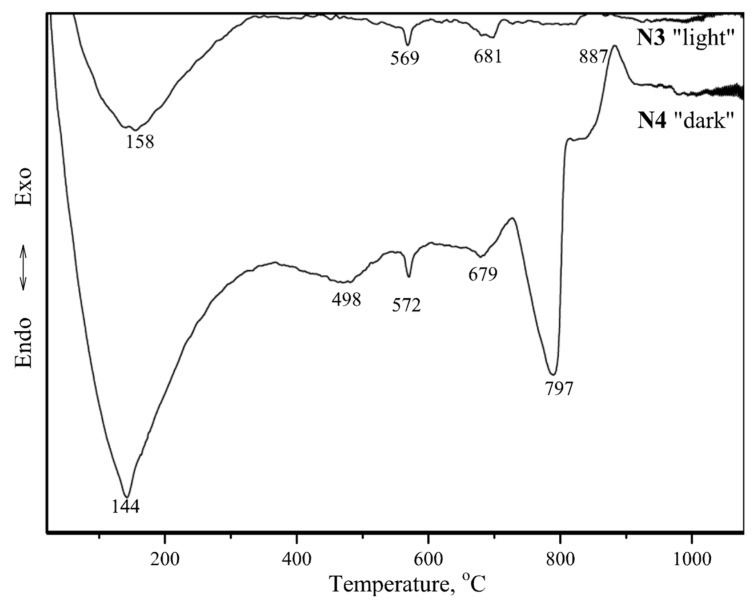
DTA curves of brick crumbles.

The DTA curves of the tested specimens (Figure 11) revealed five endothermic peaks (at approximately 140, 500, 570, 680 and 800 °C) and one exothermic peak at 887 °C for the dark-coloured ceramic brick crumbles, specimen N4, and only three endothermic peaks at approximately 160, 570, and 680 °C for the light-coloured brick crumbles, specimen N3. The endothermic peak at 800 °C in the DTA curve of specimen N4 could be attributed to the calcite decomposition, which was also identified by X-ray tests (Figure 10b). This mineral was not identified in the light-coloured brick crumbles used as mortar aggregates. Thus, the light-coloured ceramic was fired at high temperatures (the calcite present in the clay disintegrated), while the dark-coloured ceramic was apparently fired at temperatures below 800 °C (calcite was still present). Studies on the clay extracted from Lithuanian quarries [36] indicated that the local clay fired at 600 °C presented pozzolanic properties.

**Figure 12:**
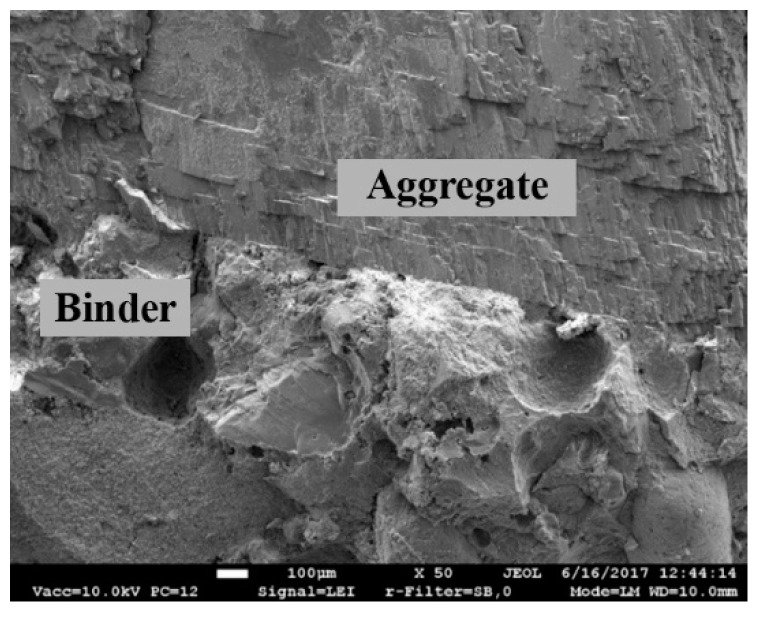
Contact zone between the brick crumble aggregate and binding material in the mortar (specimen N4).

The surfaces of crumbles investigated in this work demonstrated good pozzolanic activities and helped create good contact between the mortar and brick crumbs (Figure 12).

## 5. Conclusions

Tests on the masonry materials from retaining wall of the Gediminas Castle Hill revealed that the mortar was composed of hydraulic lime, quartz sand, and brick crumbles aggregates. The lime-to-sand mass ratio in the mortar was 1:2.3, while *FM* of sand was 2.75.

The compressive strength of the mortar reached 8–10 MPa. This was a rather high compressive strength compared to the tested strength of historical mortars from other regions. The low distributions of the compressive strength and density of the mortar observed from the test results demonstrated the high quality of the mortar and construction works. Analysis of the XRD test results revealed a high intensity peak for calcite (CaCO_3_) in the mortar specimens collected from the deepest boreholes. Microstructure tests revealed that specimens collected at the 3.5–7.7 m depth had a dense structure, whereas the specimens collected from shallower boreholes presented more pores and voids. The density and compressive strength of the mortar were found to increase with the depth of collection of the samples.

The high compressive strength of the tested mortar was attributed to the use of lime and aggregates consisting of ceramic brick crumbs. The surfaces of such crumbles demonstrated good pozzolanic activities and helped create good contact between the mortar and brick crumbs.

The results of chemical, mineral composition, and microstructure tests (XRF, XRD, and SEM/EDS) provided additional information on the materials used in the historical retaining wall of the Gediminas Castle Hill and could help select the appropriate materials for the restorations of other buildings in Vilnius dating back to the same period.

## Figures and Tables

**Table 1:** Compressive strength of mortar containing wrecked bricks (coarse aggregate concrete).

Core Sample Code	Core Sampling Depth, cm	Compressive Strength, MPa		Volumetric Density, kg/m^3^	
		Specimen	Average	Specimen	Average
**N2**	280–320	7.4	10.2 20 *	1650	1720 2.7 *
**N3**	320–380	9.2		1720	
		11.5		1780	
**N4**	390–440	7.8		1720	
**N6**	670–700	11.0		1780	
**N10**	700–730	11.8		1700	
**N12**	200–220	12.7		1710	
Note: specimens are cylinders Ø =115 mm, h =115 mm * variation, %					

**Table 2:** Properties of solid ceramic bricks.

Core Sample Code	Core Sampling Depth, cm	Compressive Strength, MPa		Water Absorption, wt.%		Volumetric Density, kg/m^3^	
		Specimen *f_i_*	Normalised ** f_bi_	Specimen	Average	Specimen	Average
**N2**	280–320	12.9	10.7	17	16 8.4 *	1870	1840 4.2 *
**N7**	200–220	18.0	14.4	15		1850	
		30.2	24.1	14		1750	
**N8**	420–450	36.4	29.5	17		1760	
		31.0	25.5	17		1830	
**N10**	700–730	22.1	18.5	15		1950	
Note: specimens are 50 × 50 × 50 mm cubes * variation, % ** the normalised compressive strength ***f_bi_*** was calculated according to LST EN 772-1 standard [23] by applying the capacity reduction factor *d* (***f_bi_*** = ***f_i_***·*d*). Average normalised compressive strength *f_b_* = 20.5 MPa (variation 33.5%)							

**Table 3:** Mortar testing results.

Core Sample Code	Core Sampling Depth, cm	Compressive Strength, MPa		Water Absorption, wt.%		Volumetric Density, kg/m^3^	
		Specimen	Average	Specimen	Average	Specimen	Average
**N1**	152–180	7.2	8.5 25 *	6.0	15.7 0.081 *	1430	1560 6.8 *
**N9**	486–530	8.1		14.4		1600	
		7.0		16.6		1450	
**N11**	731–757	9.8		14.9		1610	
		12.3		14.0		1710	
**N13**	638–658	6.7		16.1		1530	
Note: specimens are 40 × 40 × 40 mm cut cubes * variation, %							

**Table 4:** Chemical composition of mortar.

	Specimen Marking				
	N1	N2	N3	N4	N5
Oxide, wt.%					
CaO	21.58	24.32	27.42	27.06	30.67
MgO	6.67	6.00	6.76	6.55	10.76
Al_2_O_3_	4.00	5.26	4.34	4.87	4.16
SiO_2_	53.15	50.12	47.55	47.26	41.60
Fe_2_O_3_	1.03	1.20	1.33	1.68	1.38
K_2_O	0.88	1.00	1.00	1.05	0.99
Na_2_O	0.66	0.50	0.43	0.45	0.39
O	11.55	11.06	10.36	10.57	9.37
Other *	0.48	0.55	0.81	0.53	0.67

*—P_2_O_5_, SO_3_, Cl, TiO_2_, MnO, CuO.

**Table 5:** Mortar binder weight loss values obtained from TG analysis in different temperature ranges.

Specimen Marking	Weight Loss in a Certain Temperature Range (%)				Total Weight Loss (%)
	<120 °C	120–200 °C	200–600 °C	>600 °C	1000 °C
N1	1.18	1.01	4.66	8.70	15.56
N2	0.95	0.80	5.40	5.56	11.90
N3	0.96	1.08	7.59	8.93	18.56
N4	1.15	1.03	4.62	6.84	13.64
N5	0.95	0.84	4.57	8.36	14.72

**Table 6:** Average elemental composition (wt.%) of the aggregates in the tested specimens.

	Specimen Marking			
	White Inclusions		Brick Crumbles	
	N1	N2	N3 Light	N4 Dark
Chemical Element, wt.%:				
O	58.22	51.80	50.56	51.57
Si	9.60	10.36	25.34	23.86
Al	2.98	2.17	10.54	8.80
Ca	24.14	29.47	8.95	9.86
Mg	0.97	1.76	1.08	1.16
C	4.11	4.45	-	-
K	-	-	1.20	1.33
Fe	-	-	1.96	2.91
Na	-	-	0.37	0.51
Total, wt.%	100.0	100.0	100.0	100.0
